# Unfinished business after five decades of ozone-layer science and policy

**DOI:** 10.1038/s41467-020-18052-0

**Published:** 2020-08-26

**Authors:** Susan Solomon, Joseph Alcamo, A. R. Ravishankara

**Affiliations:** 1 Department of Earth, Atmospheric, and Planetary Sciences, Massachusetts Institute of Technology, Cambridge, MA 02139 USA; 2 Sussex Sustainability Research Programme, School of Global Studies, University of Sussex, Falmer, BN1 9SL UK; 3 Departments of Chemistry and Atmospheric Science, Colorado State University, Fort Collins, CO 80523 USA

**Keywords:** Atmospheric science, Environmental impact

## Abstract

The Montreal Protocol has begun to heal the Antarctic ozone hole and avoided more global warming than any other treaty. Still, recent research shows that new unexpected emissions of several chlorofluorocarbons, carbon tetrachloride, and hydrofluorocarbons, are undermining the Protocol’s success. It is time for policymakers to plug the holes in the ozone hole treaty.

The Montreal Protocol is a landmark example of policy and science teaming up to deal with a threat of global proportions. In the 1970s, the scientific community foresaw the threat posed by anthropogenic emissions to the ozone layer that protects life on Earth from harmful ultraviolet radiation. By the mid-1980s, this threat proved far worse than forecast, with a massive and unexpected springtime Antarctic ozone ‘hole’ forming due to the emissions of ozone-depleting substances (ODS), especially chlorofluorocarbons (CFCs). These scientific findings, together with clamour from civil society and governments, led to action. Negotiations under the umbrella of United Nations Environment Programme produced a political declaration in 1985, ‘The Vienna Convention’, followed in 1987 by an international agreement to act, the ‘Montreal Protocol^1^’.

The Protocol has accomplished a lot. By 2009, governments had phased out the consumption of 98% of the chemicals they agreed to in the Protocol; abundances of ODSs in the atmosphere are decreasing according to recent observations and more than 250 millions of cases of skin cancer and almost 50 millions of cases of cataracts will have been averted by the end of the century^1^. As early as 1987, the Parties emphasized that they were ‘Conscious of the potential climatic effects of emissions of these substances’ (https://treaties.un.org/doc/publication/unts/volume%201522/volume-1522-i-26369-english.pdf). Indeed, as a bonus, by 2010 the phaseout of these climate-threatening gases avoided about 15 gigatonnes (Gt) of equivalent CO_2_ emission per year, much more than the 2 Gt per year that were targeted by the Kyoto Protocol^2^.

## Much done, but much unfinished

Why has the ozone treaty been so successful? Some say it was the manageable number of sources of ODSs, whereas others highlight the actions of industry to produce practicable and profitable alternatives to ODSs. Financial assistance from OECD nations may have also encouraged all countries to participate in the treaty^1^. A keystone to the Protocol’s success has been its flexibility and the openness of the Parties to adapt to changing political climates and new knowledge provided by the Protocol’s technical panels^1^. This has led to revisions that have elevated the original aim of small reductions in CFC production to one of total global phaseout of production and consumption of these chemicals^3^.

### Re-appearing CFCs and HFCs

**Fig. 1:**
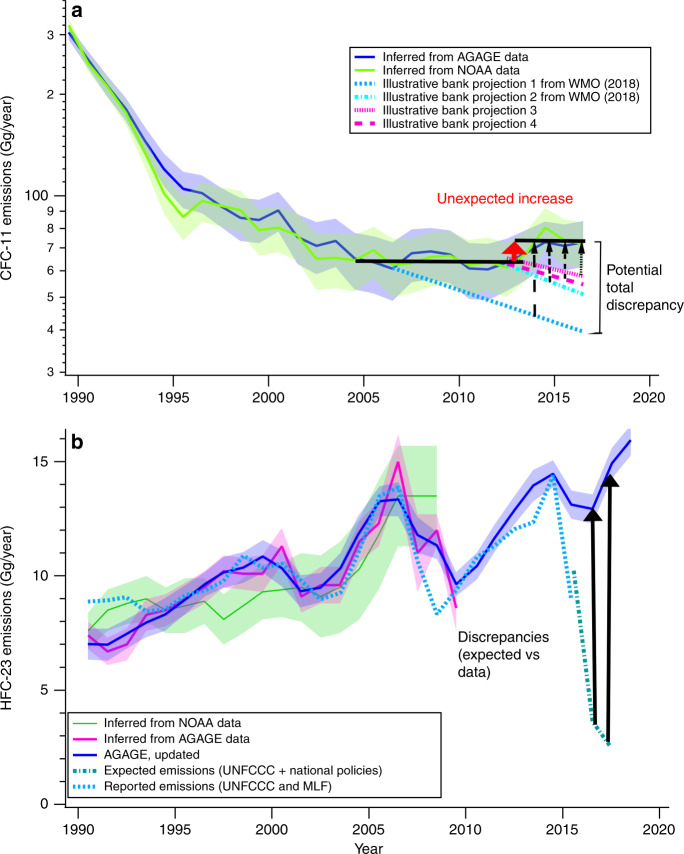
Emissions of CFC-11 and HFC-23 since 1990. **a** CFC-11 emissions with estimated 1σ error bars inferred from two different global data sets (set 1 (blue line) is from the AGAGE network, whereas set 2 (green) is from the NOAA network). Also shown are illustrative projections of emissions assuming no production outside of the Montreal Protocol and using different assumptions about bank sizes and release rates (blue and cyan lines from ref. ^21^, which assumes about 4.3–4.9% bank release rates/year, whereas pink lines include larger banks within the range of ref. ^8^, adopting 3–3.5%/year). Black horizontal lines depict averaged values for the data for 2004–2013 and for 2014–2016 as in ref. ^4^; the red arrow highlights the unexpected increase in emissions after 2013, indicating likely extra production. Black dotted and dash-dotted lines with arrows illustrate the dependence of the total discrepancy between inferred emissions from data vs. expected values from the illustrative banks. Adapted from Figs. 1–4 of ref. ^21^. **b** HFC-23 emissions with estimated 1σ error bars inferred from two different data sets (sets 2 (pink) and 3 (blue, updated from pink in ref. ^12^) are from the AGAGE network, whereas set 1 (green) is from the NOAA network, along with emissions based on national reports to the UNFCCC and Montreal Protocol Multi-Lateral Fund (MLF) through 2015 (light blue dashed line), and emissions expected based on national reports to the UNFCCC plus national policies (teal green dash-dotted line) described in ref. ^12^. Solid black lines with arrows highlight recent discrepancies between inferred emissions from data vs. expected values from international and national policies. Adapted from Figs. 2–7 of ref. ^21^, updated using ref. ^12^.

Even though extensive reductions in global ODS production have been achieved, the impact of remaining emissions loom large. Many of the remaining gases are long-lived in the atmosphere, which means that they will continue to build up even if emissions are small. In addition, disturbing evidence has emerged since the mid-2010s that the treaty is not working as well as expected. CFC-11 emissions increased by about 30% (15 ± 5 Gg/year or about 0.8 Gt CO_2_-eq/decade) from the early- to mid-2010s (Fig. 1a)^4^, which is not explainable unless there is new production in violation of the Protocol^5^. The quick detection of this problem is an important scientific success—so far, the added CFC-11 has not been enough to significantly delay the closing of the ozone hole, but continuing additions beyond 2030 would impede successful healing of the ozone layer by a decade or more^6,7^. All is not well with the other CFCs either. For example, concentrations and inferred emissions of CFC-12 and CFC-113 are decreasing more slowly than anticipated^8^ along with the unexpected new production of several minor CFCs^9–11^, raising the spectre of illicit sources of these gases as well.

Furthermore, there is the current situation of HFCs, which may undermine another great success story of the Protocol. HFCs were introduced into the economy mainly as non-ozone-depleting substitutes for CFCs, but are potent greenhouse gases. As emissions increased, governments pondered whether it was best to control them under the climate treaty, which was responsible for greenhouse gases, or under the Protocol, which was the catalyst for HFC use in the first place. The result was the Kigali Amendment of the Montreal Protocol agreed to in 2016, which levied much more stringent controls of HFCs than were possible under the current climate treaty^1^.

The story then takes another turn. HFC-23 emissions have increased more than expected in the last few years^12^, apparently falling between the cracks of successful pre-2015 actions using available technology under the climate treaty, and could undermine the formal 2020 start of Kigali (Fig. 1b)^12^. The discrepancy for 2017 is about 12 Gg/year, or about 1.5 Gt CO_2_-eq if maintained for a decade (Fig. 1b). If HFC-23 emissions do not decline this year, Kigali’s accomplishments will be called into question.

### Leaking banks of ODSs

The Montreal Protocol aims to reduce emissions of CFCs and other ODSs indirectly by controlling their production and consumption. A major gap in this strategy is that substantial emissions leak from three major CFC ‘banks’—old air conditioners, refrigerators and insulating foams. When summed, these leakages could delay the recovery of the ozone hole by as much as 6 years and add up to 10 Gt CO_2_-eq. of greenhouse gases to the atmosphere (for comparison, the European Union has pledged in the Paris Agreement to reduce its greenhouse gas emissions by a total of ~7 Gt equivalent CO_2_ between 2019 and 2030)^8^. Recent studies have emphasized that we really do not have good estimates about the sizes of the remaining banks of chemicals, or how much they are leaking; this limits attempts to quantify the extent of illicit production (Fig. 1a)^5,8^.

### Other emissions slipping through the treaty

The Montreal Protocol also does not consider ODSs used as feedstocks in making new chemicals or produced as co-products in industrial processes, as it was judged that such gases would be contained and destroyed in the manufacturing process. Neither of these judgements turned out to be correct for CCl_4_, whose production for direct uses was phased out in the Montreal Protocol but displays much larger inferred emissions than expected^13^. Recent studies indicate that carbon tetrachloride has fugitive emissions, e.g., when it is used as a feedstock to make certain HFCs and as a co-product from overchlorination in the production of cleaning agents and solvents including polychloroethylene and chloromethanes. These emissions add up to about 15 Gg/year^14^ or about 0.3 Gt CO_2_-eq/decade. Although the Protocol encourages Parties to find alternatives to these uses, it has held back in formalizing controls. Unexpectedly large emissions of CFC-113 and 113a of about 7 Gg/year (about 0.4 Gt CO_2_-eq/decade) also likely stem from leakage of feedstocks and/or intermediates. These CCl_4_ and CFC-113 emissions are of the same order as the 15 ± 5 Gg/year increase observed for CFC-11 after 2012.

### The question of N_2_O

Nitrous oxide is now the most significant ozone-depleting emission to the atmosphere, as well as the third most important greenhouse gas in terms of radiative forcing^15^. Moreover, the global emissions of nitrous oxide are accelerating^16^. Unless nitrous oxide is mitigated, it will continue to deplete the upper atmosphere ozone and undermine the gains of the Montreal Protocol.

## The next steps

The Protocol has been clearly effective in drawing down the largest culprits of ozone depletion, but now it is time to address the unfinished business.

### Toughen compliance

The basic approach to compliance of the Montreal Protocol is that Parties are expected to ‘self-report’ their own non-compliance. This report is then taken up by the Protocol’s Implementation Committee, which aims to find an ‘amicable solution’. This has worked fairly well in reducing dramatically the large amounts of production occurring through the 70s to 90s, and this short-term success is rightly celebrated. However, recent findings about renewed or excessive emissions suggest that we are reaching the limits to this approach and it’s time to consider using more stringent compliance measures contained in the Protocol.

### Eliminate feedstocks and co-products

As noted above, emissions coming from chemicals used as feedstocks for producing other chemicals, or released as co-products of a production process, are not controlled under the Protocol. Technologies exist to combat this (e.g., by separating and burning off unwanted co-products)^14^ and it is time now for Parties to negotiate stricter controls of these sources.

### Stop the leakages

Leakages from CFC banks should be halted by including policies for their safe destruction into the Protocol. This should also cover the banks of the Halons (compounds containing bromine, once used mainly in fire extinguishers), which could be recovered and destroyed. There are already precedents in international law to control materials that threaten people and the environment, e.g., the Basel Convention on Hazardous Wastes.

### Drawdown nitrous oxide

Governments have been unwilling to tackle N_2_O, because two-thirds of its anthropogenic flux comes from agriculture and some believe that the cost of mitigating N_2_O will translate into higher food costs. However, there is also evidence that emissions can be cut cost-effectively through means such as boosting the nitrogen-use efficiency of crops, which itself will bring many added benefits^17^. Therefore, it is time to finally draw down these emissions and avoid their risk to the ozone layer and climate change.

### Include environmental monitoring for effective implementation

Up to now, observational studies of ODSs and HFCs in the atmosphere have been conducted independently by the scientific community or by individual governments and have provided a critical but unofficial way to monitor progress of the Montreal Protocol. Now it is time to make these studies systematic by introducing regular environmental monitoring of CFCs, Halons, HCFCs, HFCs, CCl_4_, and N_2_O into the Protocol itself. These data are needed by the Parties to enable them to track progress and assure compliance. We note that many Multilaeral Environmental Agreements already include monitoring such as the International Convention on the Regulation of Whaling, the Convention on Long Range Transboundary Air Pollution, the Minamata Convention on Mercury, and the Stockholm Convention on Persistent Organic Pollutants^18^.

### Protect climate by protecting the ozone layer

The IPCC^19^ recently articulated the serious consequences of exceeding a global average temperature increase of 1.5 °C (we have already reached 0.9 °C), whereas studies point out that we are far off course in staying even below 2.0 °C^20^. Hence, there is unprecedented urgency in reducing as quickly as possible not only the original gases targeted by the Protocol but also all ODSs and their substitutes that contribute to global warming. These include carbon tetrachloride, CFCs-11, 12, 113, 113a, 114, and 115, the Halons, the HCFCs, the HFCs, and N_2_O. Indeed, even after the Kigali Amendment, the HFCs could still add over 20 Gt CO_2_-equivalent emissions to the atmosphere between 2020 and 2060^21^, suggesting the need for a ‘Kigali-plus Amendment’ to the Protocol, which would accelerate their planned drawdown.

Summing up, the Montreal Protocol has achieved remarkable success through its flexibility and adaptive responses to scientific and technological advances, and is hailed as the signature environmental success story of the twentieth century. It is time for it to adapt again if it is to be a twenty-first century success story.
